# Pediatric post-traumatic surgical site infection due to Eikenella corrodens: a case report

**DOI:** 10.1590/S1678-9946202668056

**Published:** 2026-08-21

**Authors:** Lanhua Guo, Xinyi Zheng, Sining Yin, Jianping Yin, Kunhua Yu, Hongjuan Zhang

**Affiliations:** 1 Shizong County People’s Hospital, Qujing, Yunnan, China; 2 The First Affiliated Hospital of Kunming Medical University, Department of Clinical Laboratory, Kunming, Yunnan, China; 3 Yunnan Province Clinical Research Center for Laboratory Medicine, Kunming, Yunnan, China. E-mail: zhanghj@ydyy.cn

**Keywords:** Children, Eikenella corrodens, Surgical site infection, MALDI-TOF MS

## Abstract

*Eikenella corrodens* is a slow-growing, fastidious gram-negative bacillus that is often overlooked in routine diagnostic procedures. We report a case of a nine-year-old girl who developed a surgical site infection five days after debridement and suturing for craniofacial trauma. Gram staining of purulent material found gram-negative coccobacilli. The isolate was presumptively identified as *Eikenella* spp. by conventional culture and biochemical testing, and later confirmed as *E. corrodens* by matrix-assisted laser desorption/ionization time-of-flight mass spectrometry. Based on the literature, amoxicillin/clavulanate was administered without formal antimicrobial susceptibility testing. Following surgical debridement and targeted antimicrobial therapy, the patient showed marked clinical improvement and achieved complete recovery within 13 days. This case highlights the importance of careful microbiological evaluation, appropriate culture conditions, and timely confirmatory identification. Close collaboration between clinicians and laboratories and access to advanced diagnostic methods or referral centers are essential for detecting uncommon pathogens.

## INTRODUCTION

*Eikenella corrodens*, a member of the HACEK group, is part of the normal flora of the oral cavity and upper respiratory tract. It is an uncommon pathogen, typically associated with bite wounds, head and neck infections, and odontogenic infections. Reports of surgical site infections caused by this organism, particularly in children, remain rare^1^. Due to its slow growing and fastidious nature, routine laboratory investigations frequently miss it. Although matrix-assisted laser desorption/ionization time-of-flight mass spectrometry (MALDI-TOF MS) has improved detection rates, diagnostic challenges persist in primary medical institutions^2^ ^), (^ ^3^. Herein, we report a pediatric case of postoperative wound infection by *E. corrodens*. The pathogen was identified early via standardized microbiological testing and laboratory-clinical collaboration. This result was confirmed by mass spectrometry at a tertiary referral center, aiming to provide a reference for identifying rare pathogens in primary care settings.

### Ethics

This single case report describes no interventional procedures. Written informed consent was obtained from the patient’s guardian, and this study was conducted in accordance with the Declaration of Helsinki.

## CASE REPORT

**Figure 1:**
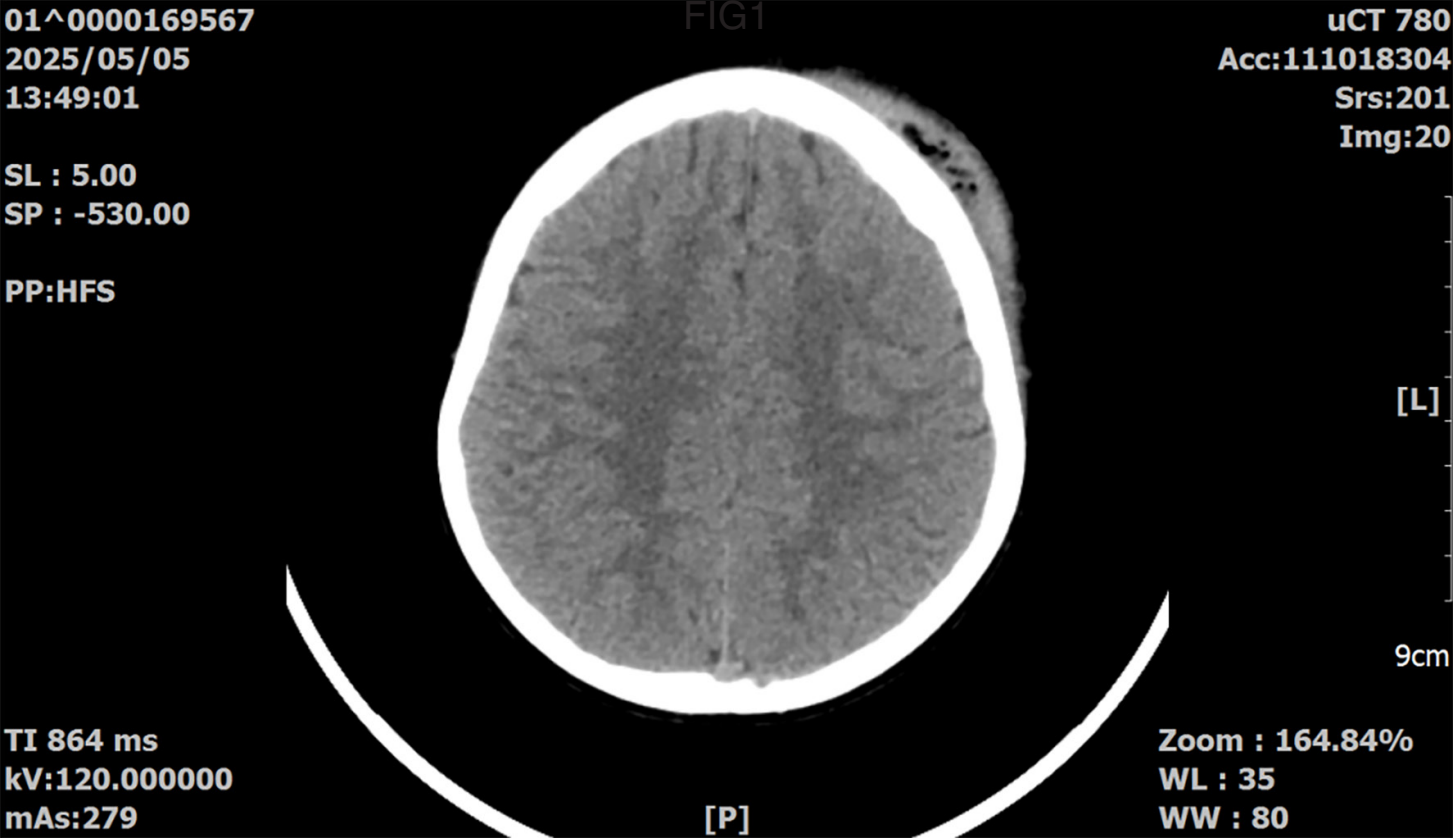
Cranial computed tomography scan showing subcutaneous air and a small hematoma in the left frontal surgical area (yellow arrows). No skull destruction, intracranial extension, or abscess formation is observed.

A nine-year-old girl presented with incisional erythema, swelling, and purulent discharge at the surgical site five days after debridement and suturing of a 3-cm left frontal wound extending to the subcutaneous tissue at a primary health care center. The patient presented with dizziness and headache but no fever, fatigue, or systemic symptoms. She had no comorbidities, immunodeficiency, history of immunosuppressive therapy, or prior antibiotic exposure. Physical examination found marked perilesional erythema and edema, with tenderness and fluctuance. Compression of the incision yielded an abundant malodorous purulent exudate. Laboratory tests showed a white blood cell count of 12.88×10^9^/L (reference range: 4.3-11.3×10^9^/L), a neutrophil count of 8.40×10^9^/L (reference range: 1.6-7.8×10^9^/L), fibrinogen of 6.14 g/L (reference range: 2-4 g/L), and D-dimer of 3.94 mg/L (reference range: <0.55 mg/L). No c-reactive protein or blood cultures were performed. Cranial computed tomography found subcutaneous air and a small hematoma at the surgical area with no evidence of skull destruction, intracranial extension, or abscess formation (Figure 1).

**Figure 2:**
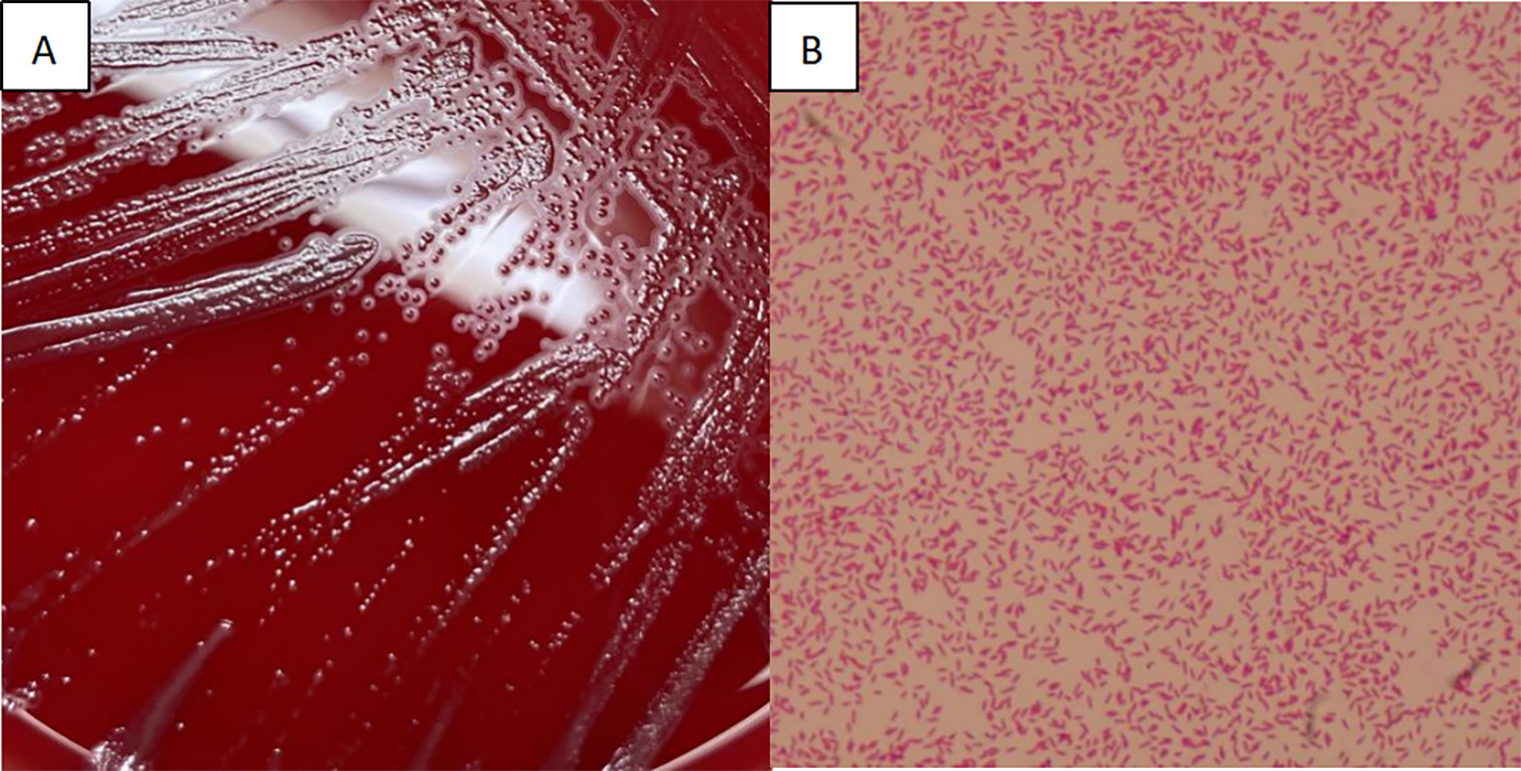
Colonial and microscopic morphology of *Eikenella corrodens*: (A) Extended incubation to 48 h resulted in larger, flat, round, translucent colonies with spreading edges and central pitting (crateriform appearance); (B) Gram stain microscopy shows gram-negative coccobacilli (×1000 magnification).

On the same day, surgical debridement and drainage were performed. An abundant foul-smelling purulent discharge was observed intraoperatively. Necrotic tissue was removed, and a drain was placed. Empirical antibiotic therapy was initiated postoperatively with ceftriaxone (50 mg/kg/day, intravenous) combined with metronidazole (15 mg/kg/day, intravenous). A deep pus specimen was collected from the surgical site for microbiological culture prior to irrigation during debridement. After 24 h of incubation at 35 °C in 5% CO_2_, small grayish-white colonies with a single morphological type were observed on Columbia blood agar (Autobio Diagnostics Co., Ltd., Zhengzhou, China), whereas no growth was detected on MacConkey agar (same manufacturer). Gram staining showed gram-negative coccobacilli. After 48 h of incubation, colonies became larger, flat, round, and translucent, with spreading edges and central pitting (Figure 2) but no additional morphotypes. Preliminary identification using microbiochemical reaction tubes (Hangzhou Binhe, Hangzhou, China) showed positive oxidase and nitrate reduction, and negative urease and glucose fermentation, consistent with *Eikenella* spp.

No antimicrobial susceptibility testing was performed. Based on published data and the known intrinsic susceptibility profile of this bacterium (susceptible to amoxicillin/clavulanate, resistant to clindamycin and metronidazole), modification of the antimicrobial regimen was recommended^4^. Accordingly, ceftriaxone and metronidazole were discontinued on postoperative day two, and intravenous amoxicillin/clavulanate (30 mg/kg/day, based on amoxicillin) was initiated. The isolate was simultaneously referred to a tertiary center for identification by MALDI-TOF MS (microflex LT/SH, Bruker Daltonics, Bremen, Germany; flexControl 3.4 with DB-12438 library), which confirmed *E. corrodens*. Intravenous amoxicillin/clavulanate was continued for 10 days. By day three of treatment, incision erythema and swelling had decreased. By day six, purulent discharge had markedly decreased. Secondary suturing was performed on day nine. By day 13, the incision had healed well, and the fully recovered patient was discharged.

## DISCUSSION

*E. corrodens* is a commensal bacterium of the oral cavity and upper respiratory tract, commonly associated with bite wounds and head and neck infections. However, its role as a causative agent of surgical site infection is rarely reported^5^, particularly in children^1^ ^), (^ ^6^. Although no history of bite injury was present in this case, frequent hand-to-mouth contact in possible contamination of the wound by oral secretions may represent a plausible route of infection. While we were unable to definitively establish this, it underscores the need for vigilance regarding infections due to oral commensals following craniofacial trauma in pediatric patients.

**Table 1:** Timeline of clinical, surgical, microbiological, and therapeutic events.

Time point	Clinical events	Surgical procedures	Microbiological findings	Antimicrobial therapy
**Day 0 (2025/04/30)**	3 cm left frontal laceration extending to subcutaneous tissue	Primary debridement and suturing at a primary care facility	Not performed	Intravenous infusion (specific drugs unknown)
**Day 5 (2025/05/05)**	Incisional erythema, swelling with foul-smelling pus, elevated inflammatory markers, subcutaneous emphysema, and hematoma on CT	Incision and drainage; deep pus specimen collected aseptically	Specimen inoculated for culture	Ceftriaxone+metronidazole
**Day 6 (2025/05/06)**	Local inflammatory symptoms	-	Small grayish-white colonies on blood agar; Gram-negative coccobacilli on staining; preliminary suspicion of *Eikenella* spp.	Ceftriaxone+metronidazole
**Day 7 (2025/05/07)**	No disease progression	-	Biochemical profile consistent with *Eikenella corrodens*; specimen referred for MALDI-TOF MS	Amoxicillin/clavulanic acid (IV)
**Day 8 (2025/05/08)**	Marked reduction of erythema and swelling	-	-	Amoxicillin/clavulanic acid (IV)
**Day 9 (2025/05/09)**	-	-	*Eikenella corrodens* confirmed by MALDI-TOF MS	Amoxicillin/clavulanic acid (IV)
**Day 11 (2025/05/11)**	Purulent discharge significantly decreased	-	-	Amoxicillin/clavulanic acid (IV)
**Day 14 (2025/05/14)**	Pain markedly relieved; no purulent discharge	Secondary suture of the incision	-	Amoxicillin/clavulanic acid (IV)
**Day 17 (2025/05/17)**	Well-healed incision, full clinical recovery	-	-	Patient discharged

The diagnostic workflow followed a stepwise, morphology-driven approach. This fastidious, slow-growing organism is easily overlooked in routine culture^3^. Although *E. corrodens* typically requires 48-72 h for visible growth, the early colony appearance at 24 h in this case may be attributable to a high bacterial load and incubation under 5% CO_2_. Early colony morphology and gram staining showing gram-negative coccobacilli provided the basis for subsequent identification. Biochemical phenotyping further narrowed the isolate to the *Eikenella* genus, with definitive species identification achieved by MALDI-TOF MS at a tertiary center.

Timely identification of *E. corrodens* directly informs clinical management. Although no antimicrobial susceptibility testing was performed in this case, previous studies indicates that *E. corrodens* is generally susceptible to amoxicillin/clavulanate and intrinsically resistant to clindamycin and metronidazole^4^ ^), (^ ^7^. This profile may conflict with common empirical regimens for postoperative wound infections^8^ ^), (^ ^9^. In this case, the antimicrobial regimen was promptly adjusted based on preliminary genus-level identification and known susceptibility patterns, avoiding potential treatment failure associated with inappropriate empirical therapy.

This study has several limitations. First, it performed no antimicrobial susceptibility testing. Antibiotic selection followed the reported intrinsic susceptibility patterns. Second, the routine diagnostic workup ignored anaerobic culture. Given the foul odor of the purulent discharge and presumed oral origin of the pathogen, polymicrobial infection with oral anaerobes cannot be excluded. The available data are unable to definitively establish *E. corrodens* as the sole pathogen rather than a component of a polymicrobial process. However, consistent pure growth of a single strain occurred at 24 and 48 h under 5% CO_2_ incubation, with no additional pathogens recovered. The infection resolved rapidly after debridement and amoxicillin/clavulanic monotherapy, supporting its role as the primary pathogen. Third, no fungal culture was performed. Finally, as a single case report, the generalizability of these findings are limited.

In summary, this case underscores the importance of considering rare oral commensal pathogens in the differential diagnosis of pediatric post-traumatic surgical site infections. A stepwise diagnostic approach, combined with timely antimicrobial adjustment guided by microbiological findings, is critical for achieving favorable clinical outcomes.

## CONCLUSION

*E. corrodens* is a rare causative pathogen of surgical site infection, especially in pediatric patients following traumatic injury. Its pathogenesis may be linked to contamination by oral flora, warranting clinical awareness. As a slow-growing and fastidious organism, it may be easily missed in routine culture. Nevertheless, even in resource-limited settings, accurate diagnosis and effective management of infections caused by rare pathogens can be achieved with standardized diagnostic and therapeutic protocols, along with close collaboration between primary care facilities and referral laboratories.

## Data Availability

The complete anonymized dataset supporting the findings of this study is included within the article itself.
